# Social Determinants of Learning in Medical Education: Application to Neurology and Neurosciences Education

**DOI:** 10.1212/NE9.0000000000200134

**Published:** 2024-06-10

**Authors:** Anita V. Shelgikar, Cornelius A. James

**Affiliations:** From the University of Michigan, Ann Arbor.

People love to say, “Give a man a fish, and he'll eat for a day. Teach a man to fish, and he'll eat for a lifetime.” What they don't say is, “And it would be nice if you gave him a fishing rod.” That's the part of the analogy that's missing. —Trevor Noah^1^

If each student receives the same curricular tools—stethoscopes, microscopes, and computers—are they equally positioned to succeed in medical school? What factors disproportionately affect some students despite medical schools' best intentions to level the playing field for all?

Medical educators need to identify, understand, and address the noncurricular social determinants of learning (SDOL)^2^ that determine educational equity. SDOLs include individual (physical health, psychosocial health, physical environment, social environment, economic stability, self-motivation) and institutional (culture and activities, faculty, curriculum, environment, administration, campus life, students) determinants of academic success. Tenets of diversity, equity, and inclusion intersect the individual and institutional SDOLs. As we look to diversify the physician workforce, we need to define medical schools' responsibility in addressing factors that are simultaneously related to students' human existences and their learning.

Students' medical school experiences are influenced by their peer community, faculty relationships, academic climate, meaningful engagement, mentoring, inclusion, safety, and physical space.^3^ Each student is the culmination of personal experiences that may affect their professional trajectories during and beyond medical school. Consider, for example, the intersection of economic stability and self-identity: Individuals from groups underrepresented in medicine have the highest student loan debt upon graduation from medical school.^4^ Economic instability may lead to forced decisions (e.g., leaving medical school, chosen specialty) rather than decisions guided by interest or passion.^5^ Furthermore, medical students' motivation is proportional to their learning quality and performance, and medical educators affect their students' motivation to learn, making it critical for neurology and neuroscience educators to understand how these determinants affect students' learning.^6^

As educators we need to expand the biopsychosocial approach to medical student education and intentionally include SDOLs. Dedicated student and faculty development can train learners and educators on how to recognize and address SDOL in their learning environments (Table 1). Student-school and student-student partnerships will help cultivate systems that (1) allow students to seek assistance through mechanisms that appropriately protect their privacy, (2) facilitate a culture in which students, staff, and faculty feel empowered to seek support, and (3) allow educators to understand and address the root cause(s) when a student is struggling academically. Table 2 presents how neurology educators and neuroscience educators may use the SDOL framework in their respective areas.

**Table 1:** Opportunities for Trainee and Faculty Development About the SDOL

Objectives	Activities to consider
Intent: To increase awareness of SDOL	
Provide an overview of SDOL	Develop asynchronous online learning modules to introduce the concept of SDOL and how these apply to medical education
Identify strategies used by other institutions/programs to identify and/or address SDOL among students	Host journal clubs dedicated to literature about • Processes used by a program or institution to identify SDOL that may influence a trainee's performance • System-level strategies implemented within a program or institution to address SDOL among learners
Intent: Create program-level changes to incorporate SDOL into educational processes	
Incorporate SDOL into holistic review of trainee performance	Educate members of the CCC • Hold a CCC retreat to review current assessment methods and identify areas that would be amenable to inclusion of SDOL • Adapt assessment forms of trainee performance to include discussion of SDOL (e.g., Include a question such as “Is the physical environment in some way impeding the student's learning?”)
Recognize opportunities to embed SDOL into the program's structure	Include SDOL as a regular discussion item during program evaluation • Do all learners in the program have the resources they need to optimize their learning? If not, what different resources can the program provide? • Do program faculty understand how to assess for SDOL in a nonjudgmental way? If not, designate faculty meetings and/or grand rounds dedicated to SDOL
Intent: Implement institution-level changes to make the learning environment more inclusive and responsive to SDOL	
Understand how to use the SDOL framework to customize curricular components	Convene medical school and graduate medical education leadership to discuss cross-cutting opportunities to incorporate SDOL • Identify administrative policies and procedures that may adversely affect cohorts of students (e.g., leave of absence policies that may cause undue stress for those with economic instability) • Hold a symposium, inclusive of outside speakers and breakout sessions, to identify best practices and the institutional resources that would be needed to implement these practices at the institution • Facilitate collaboration between the offices of Financial Aid, Health Equity, and Well-Being to pursue a well-rounded examination of system-level factors that influence (or are influenced by) SDOL
Support student growth and success using a SDOL framework	Create workgroups with student-faculty partnerships • Address aspects of campus life that could be more inclusive of students from historically marginalized backgrounds • Ongoing re-evaluation of the climate of the institution/program to identify opportunities for improvement

Abbreviations: CCC = clinical competency committee; SDOL = social determinant of learning.

**Table 2:** Examples of How to Apply the SDOL Framework in Neurology and Neuroscience Education

Case example	Related SDOL(s)	Action to address SDOL	Potential partners to help address SDOL
Neuroscience education: You are the faculty lead for the first-year medical students' neuroscience course. You inform the class about an unfunded summer research opportunity in your laboratory. A student expresses interest but later reconsiders because they cannot afford rent if they accept an unpaid position. You consider how your laboratory can help promote educational equity	*Individual SDOL(s):* Economic stability *Intersecting SDOL(s):* Diversity, equity, inclusion *Institutional SDOL(s):* Culture and activities Administration	Seek out mechanisms to secure funding for the summer research position to make the opportunity accessible to more students	• Institutional work-study office and/or office of financial aid • Specialty and subspecialty foundations that fund scientific discovery • Institutional development (fundraising) office
Neurology clinical learning environment: You are a chief resident in a neurology program, and you have noticed that photos honoring individuals in the departmental office and the neurology units lack diversity In addition, the neurology hospital services (i.e., stroke) are named to honor in individuals, but appear to lack diversity You have concerns that these factors could impact the sense of belonging amongst current students. You are also concerned about student recruitment and retention, and would like to consider how to make the program more welcoming and inclusive for all	*Individual SDOL(s):* Physical environment/community Psychosocial health *Intersecting SDOL(s):* Diversity, equity, inclusion *Institutional SDOL(s):* Environment Campus life	Identify individuals from diverse backgrounds that have made significant contributions to the department, program, or the field of neurology • Include photos of these individuals in offices and in the hospital • Consider renaming or co-naming the neurology services	• Department chair • Residency program director • Section leads or chiefs • Office of health equity and inclusion
Neurology clinical learning environment: You are a newly appointed faculty member in a neurology residency program. After a few months on the inpatient ward and consult services, you realize that many students and residents cannot afford ophthalmoscopes and thus omit bedside fundoscopy. The post-rotation assessment form indicates that you should downgrade learners' ratings if they do not include fundoscopy as part of their neurologic exam	*Individual SDOL(s):* Economic stability *Intersecting SDOL(s):* Diversity, equity, inclusion *Institutional SDOL(s):* Curriculum Faculty	Work with the program and departmental leadership to purchase a set of shared ophthalmoscopes for students and residents to use Provide faculty development around learners' financial barriers and that students' ability to purchase tools (e.g., ophthalmoscope) is not a reflection of their professionalism, medical knowledge, or patient care	• Residency program director • Neurology clerkship director • Neurology department chair and administrator • Office of medical student education • Institutional and departmental development (fundraising) offices
Neurology clinical learning environment: You are the attending physician on the neurology inpatient service. After rounds the medical students and residents start talking about their after-work plans. You overhear one student mention that they spend most of their evenings in the hospital cafeteria because their home is unheated. You recognize that the student's housing situation can directly influence their learning experience on the wards	*Individual SDOL(s):* Physical environment/community Psychosocial health Social environment/community *Intersecting SDOL(s):* Diversity, equity, inclusion *Institutional SDOL(s):* Environment Students Campus life	Inform medical school leadership, while trying to maintain the student's agency and privacy	• Office of Student Affairs • Medical school counselors • Community-based housing organizations
Neurology/neuroscience mentoring: You are a faculty career advisor for medical students who are considering neurology as their chosen specialty. During your meetings with students, you realize that many students would like to pursue a visiting elective at another school defer the opportunity due to associated costs (e.g., application fee, housing, travel between clinical training sites). You are concerned that this economic factor may disadvantage students during the residency application process	*Individual SDOL(s):* Self-motivation Economic stability *Institutional SDOL(s):* Diversity, equity, inclusion Curriculum Students	Work with medical school and departmental leadership to identify funding sources for medical students use towards visiting electives	• Office of Student Affairs • Medical school counselors • Office of financial aid • Department chair and administrator • Institutional and departmental development (fundraising) offices
Neurology/neuroscience mentoring: You are the faculty mentor for a neurology student's research project. The student sought out this research opportunity and has worked tirelessly on the project. An abstract you submitted together was accepted for an oral presentation at a national meeting, and you encourage the student to give the presentation. The student would welcome the opportunity but due to mobility impairment is not sure they will be able to safely navigate the meeting space	*Individual SDOL(s):* Self-motivation Physical health Diversity, equity, inclusion *Institutional SDOL(s):* Institution's culture and activities	Create or use an existing departmental or institutional recognition program to highlight student engagement in the research activities Identify mechanisms to secure safe, accessible accommodations for the student present their research	• Organization that is hosting the meeting • Departmental institutional and faculty

Abbreviation: SDOL = social determinant of learning.

An aspiration is that the SDOL framework may facilitate addressing SDOLs before they begin to affect a student's academic performance. With an understanding of SDOL, we can work to support diversity, equity, and inclusion in medical education and equip all medical students with the tools needed to thrive throughout their training and careers.

## Appendix Authors

**Table:**

Name	Location	Contribution
Anita V. Shelgikar, MD, MHPE	University of Michigan, Ann Arbor	Drafting/revision of the manuscript for content, including medical writing for content; major role in the acquisition of data; study concept or design; analysis or interpretation of data
Cornelius A. James, MD	University of Michigan, Ann Arbor	Drafting/revision of the manuscript for content, including medical writing for content; major role in the acquisition of data; study concept or design; analysis or interpretation of data
